# Predictors of Hepatocellular Carcinoma Early Recurrence in Patients Treated with Surgical Resection or Ablation Treatment: A Single-Center Experience

**DOI:** 10.3390/diagnostics12102517

**Published:** 2022-10-17

**Authors:** Mauro Giuffrè, Enrico Zuliani, Alessia Visintin, Paola Tarchi, Paola Martingano, Riccardo Pizzolato, Deborah Bonazza, Flora Masutti, Rita Moretti, Lory Saveria Crocè

**Affiliations:** 1 Department of Medical, Surgical and Health Sciences, University of Trieste, 341349 Trieste, Italy; 2 Liver Clinic, University Hospital of Trieste (Azienda Sanitaria Giuliano-Isontina), 34149 Trieste, Italy; 3 Surgical Clinic, University Hospital of Trieste (Azienda Sanitaria Giuliano-Isontina), 34149 Trieste, Italy; 4 Diagnostic and Interventional Radiology, University Hospital of Trieste (Azienda Sanitaria Giuliano-Isontina), 34149 Trieste, Italy; 5 Anatomic Pathology and Histology, University Hospital of Trieste (Azienda Sanitaria Giuliano-Isontina), 34149 Trieste, Italy

**Keywords:** HCC, early recurrence, tumor nodule dimension, surgical resection, ablation treatment

## Abstract

**Introduction:** Hepatocellular carcinoma (HCC) is the sixth most diagnosed malignancy and the fourth leading cause of cancer-related death worldwide, with poor overall survival despite available curative treatments. One of the most crucial factors influencing survival in HCC is recurrence. The current study aims to determine factors associated with early recurrence of HCC in patients with BCLC Stage 0 or Stage A treated with surgical resection or local ablation. **Materials and Methods:** We retrospectively enrolled 58 consecutive patients diagnosed with HCC within BCLC Stage 0 or Stage A and treated either by surgical resection or local ablation with maximum nodule diameter < 50 mm. In the first year of follow-up after treatment, imaging was performed regularly one month after treatment and then every three months. Each case was discussed collectively by the Liver Multidisciplinary Group to decide diagnosis, treatment, follow-up, and disease recurrence. Variables resulting in statistically significant difference were then studied by Cox regression analysis; univariately and then multivariately based on forward stepwise Cox regression. Results are represented in hazard ratio (H.R.) with 95% confidence interval (C.I.). **Results:** There was no statistically significant difference in recurrence rates (34.8 vs. 45.7%, log-rank test, *p* = 0.274) between patients undergoing surgical resection and local ablation, respectively. Early recurrence was associated with male gender (HR 2.5, 95% C.I. 1.9–3.1), nodule diameter > 20 mm (HR 4.5, 95% C.I. 3.9–5.1), platelet count < 125 × 103 cell/mm^3^ (HR 1.6, 95% C.I. 1.2–1.9), platelet-lymphocyte ratio < 95 (HR 2.1, 95% C.I. 1.7–2.6), lymphocyte-monocyte ratio < 2.5 (HR 1.9, 95% C.I. 1.4–2.5), and neutrophil-lymphocyte ratio > 2 (HR 2.7, 95% C.I. 2.2–3.3). Discussion and Conclusions: Our results are in line with the current literature. Male gender and tumor nodule dimension are the main risk factors associated with early HCC recurrence. Platelet count and other combined scores can be used as predictive tools for early HCC recurrence, although more studies are needed to define cut-offs.

## 1. Introduction

Hepatocellular carcinoma (HCC) is the sixth most diagnosed malignancy and the fourth leading cause of cancer-related death worldwide, and according to recent estimations, by 2025, more than a million people will develop HCC annually [1,2]. The global incidence of HCC is highly variable due to differences in risk factors associated with chronic liver disease, with 72% occurring in Asia alone [2]. Globally, 54% of HCC is caused by chronic hepatitis B virus (HBV) infection and 31% by hepatitis C virus (HCV) infection, with the remaining portion associated with alcoholic liver disease (ALD) and non-alcoholic liver disease (NAFLD), with the latter being on the rise as contributors to HCC development [3,4,5]. Unfortunately, HCC has a median overall survival from 6 to 20 months after diagnosis, with a two-year survival of less than 50% and five-year survival of only 10% if left untreated [6,7,8,9]. The Barcelona Clinic Liver Cancer (BCLC) classification, based on tumor burden, liver function, and patient performance status, is the most utilized staging system that provides a rationale for clinical decisions [10]. For example, patients with BCLC Stage 0 or Stage A can be treated with more radical approaches (i.e., curative treatments), such as liver transplantation, surgical resection, or local ablation, either by microwave ablation (MWA) or radiofrequency ablation (RFA) [11]. In these cases, surgical treatment offers a five-year survival rate of 70–80% with low perioperative mortality <5% [12,13]; local ablation reaches a five-year survival rate of 50–70% [14,15]. One of the most crucial factors influencing survival is HCC recurrence: even after potentially curative treatments, recurrent HCC develops in 30–50% and 50–70% of patients within the first two years and five years of follow-up, respectively [16,17,18,19]. Many factors affect the risk of post-treatment recurrence, including tumor size > 3 cm, satellite tumor, tumor encapsulation, proximity to large vessels and their invasion (either macroscopic or microscopic), partial necrosis, concurrent liver cirrhosis, serum a-fetoprotein > 400 ng/L, and antiviral treatment, or viral etiology [20,21,22]. Early recurrence mainly results from undetected micrometastases rather than de novo tumors arising in a microenvironment predisposed to carcinogenesis [16]. Many studies focus on novel biomarkers or other histological characteristics which can predict the early recurrence of HCC [23,24,25,26,27].

## 2. Materials and Methods

### 2.1. Population and Study Design

In this study, we evaluated 130 consecutive patients diagnosed with HCC and referred to the Liver Clinic of the University Hospital of Trieste [9] from 1 January 2014 to 31 December 2021 (*n* = 130). This study complied with the Declaration of Helsinki and was performed according to ethics committee approval. Informed consent was obtained from each patient before inclusion in the study.

Radiological features of liver lesions were described according to the Liver Imaging Reporting and Data System (LI-RADS) criteria [28]. In contrast, the response to treatment was evaluated according to the modified Response Evaluation Criteria in Solid Tumors (mRECIST) for HCC [29].

Data were collected from digitalized medical charts of patients and subsequently analyzed from an anonymous database. To be included in the study, patients had to be staged as BCLC Stage 0 or Stage A and selected for treatment by surgery or local ablation (MWA or RFA). We excluded: (1) patients who underwent surgical resection with proven histological microvascular invasion or undetected satellite nodules; (2) patients with macrovascular invasion disregarding the treatment type; and (3) patients with baseline imaging with >2 nodules with LI-RADS score = 5 nodule ± other nodules with a LI-RADS score ≥ 3 to exclude de-novo carcinogenesis as a confounding factor. From the original 130 patients, 59 matched the inclusion and exclusion criteria, with one patient lost to follow-up after treatment. After treatment, each patient underwent clinical and radiological follow-up consisting of abdominal computed tomography (CT) scans at 1, 3, 6, 9, and 12 months. The follow-up was censored at 12 months after treatment. Radiological images were evaluated collectively by the Liver Multidisciplinary Team, composed of hepatologists, radiologists, surgeons, and pathologists.

The following data were collected for each patient: sex, age at treatment, and etiology of underlying liver disease. Regarding patients’ HCC status, the following parameters were recorded: number of nodules, maximum diameter of the target lesion (mm), and BCLC stage. In terms of laboratory data, the following were collected the week before treatment: aspartate aminotransferase (AST), alanine aminotransferase (ALT), g-glutamyl transferase (GGT), alkaline phosphatase (ALP), total bilirubin, serum albumin, platelet count, international normalized ration (INR), α-fetoprotein (AFP), complete blood count, and serum creatinine. The following scores were calculated for each patient: platelet/lymphocyte ratio (PLR), neutrophils/lymphocytes ratio (NLR), lymphocytes/monocytes ratio (LMR), Child–Pugh score, and MELD score.

### 2.2. Statistical Analysis

The Shapiro–Wilk test was performed to verify the normal distribution of variables. According to the test results, continuous variables were reported as medians (Quartile 1; Quartile 3), while discrete variables were reported as the number and proportion of subjects with the characteristic of interest. Patients were firstly categorized into two groups according to treatment (Group 1 = surgery; Group 2 = ablation). Intergroup differences were investigated using the Mann–Whitney U test for continuous variables and Pearson’s chi-square test for discrete variables [30]. The recurrence-free interval between the two groups was studied with a log-rank test and represented graphically with Kaplan–Meier estimator [31,32].

Subsequently, patients were categorized into two groups according to recurrence (Group 3 = recurrence; Group 4 = without recurrence). Intergroup differences were investigated using the Mann–Whitney U test for continuous variables and Pearson’s chi-square test for discrete variables. Variables resulting in statistically significant difference were then studied by Cox regression analysis [33], univariately and then multivariately based on forward stepwise Cox regression, with the best model chosen according to Bayesian information criterion (BIC) [34]. Results are represented in hazard ratio (H.R.) with 95% confidence interval (C.I.). Before being entered into the regression model, discriminative capabilities of continuous variables were investigated through the area under the receiver-operating characteristic curve (AUROC). Accordingly, these variables were categorized into two groups according to Youden’s Index [35], except for maximum nodule diameter, which also considered values of 30 and 40 mm.

For all analyses, two-sided statistical significance was defined as *p* < 0.05. Data were analyzed using SPSS (Statistical Package for Social Science) version 26.0 (IBM SPSS Statistics for MAC OS. Armonk, NY, USA: IBM Corp.).

## 3. Results

### 3.1. Patients Characteristics

**Figure 1:**
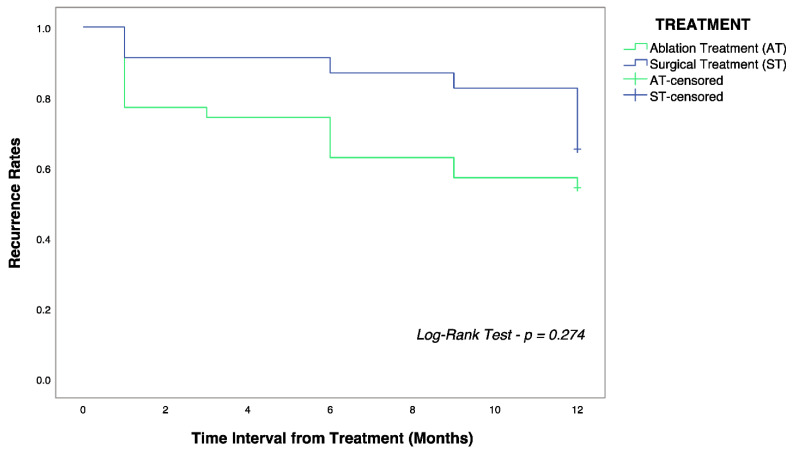
Kaplan–Meier curves showing overall recurrence-free survival in the surgery (blue) and ablation (green) group. The log-rank test did not show statistically significant differences (*p* = 0.274) between the two groups of patients.

As reported in Table 1, we enrolled 48 males (82.8%) and ten females (17.2%), with a median age of 70 (65;77) years. 39.6% underwent surgical treatment, whereas 60.4% underwent ablation treatment. Patients undergoing surgery were younger (66 vs. 72 years, *p* = 0.003), with larger median nodules diameter (25 vs. 20 mm, *p* = 0.003), higher platelet count (171 vs. 109 × 103 cells/mm^3^, *p* = 0.003), and longer median interval of recurrence from treatment (10.5 vs. 2 months, *p* = 0.016), despite no statistically significant difference in recurrence rates (34.8 vs. 45.7%) as also shown by Figure 1 (log-rank test, *p* = 0.274).

### 3.2. Analysis on HCC Recurrence

Patients were then divided according to HCC recurrence. As shown in Table 2, patients with HCC recurrence were mostly men (91.7% vs. 76.5%, *p* = 0.032), with larger median nodule diameter (33 vs. 18 mm, *p* < 0.0001), lower median platelet count (111.5 vs. 143 × 103 cells/mm^3^, *p* = 0.03), lower median PLR s (92 vs. 103.5, *p* = 0.026), higher LMR (3.2 vs. 2.6, *p* = 0.041), and lower NLR (1.9 vs. 2.6, *p* = 0.043). Discriminative ability of statistically significant different continuous variables was investigated through AUROC analysis: nodule maximum diameter, AUROC 0.86 (95% C.I. 0.76–0,96, *p* < 0.001); platelet count, AUROC 0.66 (95% C.I. 0.52–0.80, *p* = 0.013); NLR, AUROC 0.66 (95% C.I. 0.51–0.80, *p* = 0.025); LMR, AUROC 0.63 (95% C.I. 0.48–0.77, *p* = 0.021); PLR, AUROC 0.62 (95% C.I. 0.47–0.76, *p* = 0.032). Their respective cut-offs, chosen according to Youden’s Index, are reported in Table 3, where they are computed with statistically significant different continuous variables for Cox regression analysis.

## 4. Discussion

In this retrospective longitudinal study, fifty-eight patients with HCC were followed up for 12 months after being treated by surgical resection or local ablation. It is crucial to highlight that there is no universally adopted definition for “early recurrence”, with some authors setting the cut-off at eight months after treatment [36] and others at 24 months after treatment [37]. We arbitrarily defined the early recurrence cut-off at twelve months after treatment because, after the first year from treatment, follow-up CT scan intervals are less frequent, thus resulting in more challenges to precisely date the recurrence period.

### 4.1. Surgical Resection vs. Local Ablation

No statistically significant difference in recurrence rates was found between patients undergoing surgery (34.8%) and those undergoing local ablation (45.7%). However, patients undergoing surgical resection had a longer median recurrence-free interval than those who underwent local ablation (10.5 vs. 2 months, *p* = 0.0016). A comparison of resection and ablation for HCC based on a Japanese nationwide survey, including patients who had no more than three tumors (≤3 cm), demonstrated that the recurrence risk was lower in patients undergoing surgical resection when compared to ablation [38]. On the contrary, a randomized controlled trial demonstrated worse recurrence-rates survival in patients undergoing surgery, only at 24 months, without any difference at 12 months [38]. Other trials confirm this trend [39,40,41]. Kutlu et al. did not find any difference between the two techniques in terms of recurrence for nodules ≤ 3 cm, but better outcomes in terms of survival and recurrence-free-interval for surgery techniques, in particular when HCC nodules were between 3 and 5 cm diameter [42]. This data is confirmed by a recent multicenter clinical study that showed comparable and recurrence-free survival between the two techniques [43]. The abovementioned evidence is in line with our results, considering that 75% of the patients included had nodules ≤ 3 cm, with patients undergoing surgery presenting a slightly larger median nodule maximum diameter. Also, the absence of micro- and macro-vascular invasion makes the two treatment groups comparable to the baseline recurrence risk, providing similar outcomes in the first twelve months after treatment. At the same time, it is worth highlighting that patients undergoing local ablation were older and with lower platelet count when compared to the surgical group, highlighting a possible selection and worst outcomes which were not evident in our cohort of patients

### 4.2. Male Gender

HCC is sexually dimorphic in both rodents and humans, with a significantly higher incidence in males, with an average male-to-female ratio of 2–4:1 across studies [44]. Both sex hormones and inflammatory response have been implicated in this gender disparity, with studies in zebrafish showing that male individuals tend to develop more advanced multi-nodular tumors compared to female individuals [45]. In our cohort of patients, male individuals were at higher risk for early HCC recurrence with an H.R. of 2.5 (95% C.I. 1.9–3.1). Many studies have reported higher morbidity and worst survival outcomes in male patients with HCC [46]. Liang et al. showed that the male gender has an H.R. of 1.48 (95% C.I. 1.08–2.02) for early recurrence after surgical resection [47], Yang et al. reported that the male gender has an H.R. of 2.46 (95% C.I. 1.60–3.79) for early recurrence after ablation treatment [48], while Bae et al. [49] found that male gender has an H.R. of 1.35 (95% C.I. 1.12–1.63) for early recurrence after curative treatment. Nevertheless, other studies did not report any significant difference in early, gender-related HCC recurrence [50,51,52]. Despite no precise data, it can be assumed that the same hormonal factors promoting tumorigenesis might induce even HCC recurrence.

### 4.3. Tumor Dimension

Post-treatment recurrence of HCC is closely related to tumor characteristics, such as tumor size, capsular integrity, differentiation degree, and presence of vascular invasion, with larger tumor size confirmed as a critical parameter affecting early recurrence [53]. As shown in Table 3, tumor nodule > 20 mm is associated with an increased risk of HCC recurrence, HR 4.5 (95% C.I. 3.9–5.1), with a further increase in recurrence risk for nodules > 30 and > 40 mm. According to current evidence, HCC nodules > 50 mm are the ones at higher risk for recurrence [36,54]. However, even smaller nodule dimensions should be taken into consideration. Shirabe et al. found that patients with early recurrence have median nodule diameters > 22 mm [55]; Zhu et al. reported that patients with tumor diameters > 2.6 cm had a 4.77 times higher risk for recurrence [56]. Accordingly, other studies have reported similar data for larger nodules [36,54,57], as shown by Lee et al., where nodule diameter > 30 mm had an H.R. of 2.03 (95% C.I. 1.49–2.77) and nodule diameter > 50 mm had an H.R. of 2.26 (95% C.I. 1.66–2.97) [58].

### 4.4. α-Fetoprotein

AFP levels are considered unsuitable for HCC screening, surveillance, and diagnosis because of their low sensitivity and specificity [59]. Thus, only values > 1000 ng/mL appear to be significant for HCC recurrence [60,61]. Nevertheless, AFP levels > 200 ng/mL were associated with a 3.32-fold increase in the probability of HCC recurrence [62], and AFP levels > 400 ng/mL were associated with a 2.21-fold increase in the probability of HCC recurrence [63]. Our study did not find a significant correlation between AFP levels and HCC recurrence [59], in line with the current literature.

### 4.5. Platelet Count

Growing evidence has highlighted the role of platelets in favoring HCC growth and dissemination. Several growth factors and angiogenetic molecules secreted by platelets (i.e., platelet-derive growth factor, vascular endothelial growth factor, endothelial growth factor, and serotonin) are directly related to tumor progression and neo-angiogenesis [64]. Platelets were also found involved in tumor spread, by increasing endothelium permeability, favoring neoplastic cells extravasation and survival in the bloodstream [64]. Thrombocytopenia, whose pathogenesis is multifactorial in advanced liver disease, is currently incorporated in models aimed at predicting the development of HCC and in decision-making models for the selection of HCC treatment [65], given the possible risk of worse portal hypertension and outcomes [66,67]. As a matter of fact, the results of a recent meta-analysis suggested that thrombocytopenia seems to be associated with reduced overall (HR = 1.41, 95% CI = 1.14–1.75) and recurrence-free survival (HR = 1.44, 95% CI = 1.13–1.83) [68], independently from the treatment used, which is in line with our results, thus explaining why a combined score that includes platelet (i.e., PLR) was discovered to predict the risk of HCC recurrence [69]. Elevated PLR values were associated with poor prognosis in patients with HCC. In particular, high pretreatment PLR values were associated with both poor overall (HR = 1.73; 95%CI = 1.46–2.04; *p* < 0.001) and disease-free survival (HR = 1.30; 95%CI = 1.06–1.60; *p* = 0.01) [70].

### 4.6. Lymphocyte-Monocyte Ratio

Regarding combined scores, recent studies have demonstrated that LMR is valid for a significant prognostic oncological factor [71,72]. In patients with HCC, a low LMR is associated with worse outcomes and an increased risk of recurrence [73]. However, studied LMR cut-offs are highly variable in each study, thus determining a low reproducibility [49].

### 4.7. Neutrophil-Lymphocyte Ratio

The NLR is one of the most widely studied inflammatory markers in recent years [74]. The relationship between elevated NLR and worse outcomes in HCC is complex and has already been explored elsewhere [75]. A recent meta-analysis on HCC patients found that elevated NLR values (ranging from 3 to 4) were associated with poor overall survival (H.R. = 2.17; 95% C.I. 1.41–3.34) and worse disease-free survival (H.R. = 2.36, 95% C.I. 1.54–3.60). Our study reflects this trend. However, our cut-off value was set lower to best fit our population, with only around 25% of patients having NLR values > 3 [76].

### 4.8. Strengths and Limitations

Regarding the critical aspect of our study, the main limitations are related to the small number of patients and the study’s retrospective nature leading to possible selection bias and the impossibility to conduct sub-group analysis within the two main groups. On the other hand, only one patient was lost at follow-up, and each patient was strictly followed during the first 12 months after treatment. Also, each enrolled patient was reviewed by the multidisciplinary liver teams, having a collective consensus on HCC diagnosis, treatment options, and recurrence.

## 5. Conclusions

Our results are in line with the current literature. Male gender and tumor nodule dimension are the main risk factors associated with early HCC recurrence. Platelet count and other combined scores (such as PLR, LMR, or NLR) can be used as a predictive tool for early HCC recurrence, although more longitudinal studies are needed to define cut-offs.

## Figures and Tables

**Table 1:** Clinical, biochemical, and staging characteristics of the enrolled population (*n* = 58). Abbreviations: BCLC: Barcelona Clinic Liver Cancer; AST: aspartate aminotransferase; ALT: alanine aminotransferase; GGT: γ-glutamyl transferase; ALP: alkaline phosphatase; INR: international normalized ration; AFP: α-fetoprotein; PLR: platelet/lymphocyte ratio; LMR: lymphocytes/monocytes ratio; NLR: neutrophils/lymphocytes ratio; NS: not significant; NA: not applicable.

Variables	Altogether N = 58	Surgery N = 23	Ablation N = 35	Significance
Gender, Male	48 (82.8%)	18 (78.3%)	30 (85.7%)	NS
Age, years	70 (65; 77)	66 (61.5; 71)	72 (69.5; 79)	*p* = 0.003
Etiology, Viral	27 (46.6%)	13 (56.5%)	14 (40%)	NS
Presence of Liver Cirrhosis	51 (87.9%)	19 (82.6%)	31 (88.6%)	NS
Number of Nodules, number	1 (55, 94.2%) 2 (3, 6.8%)	1 (23, 100%) 2 (0, 0%)	1 (32, 91.4%) 2 (3, 8.6%)	NA
Nodule Maximum Diameter, mm	22 (15; 30)	25 (20; 44)	20 (14.5; 26)	*p* = 0.003
BCLC				
Stage 0 Stage A	28 (48.3%) 30 (51.7%)	9 (39.1%) 14 (60.9%)	19 (54.3%) 16 (45.7%)	NS
AST, IU/L	25.5 (22; 37)	24 (21.5; 39)	26 (22; 36.5)	NS
ALT, IU/L	22 (17; 35)	21 (17; 38)	23 (16; 29.5)	NS
ALP, IU/L	92 (75; 112)	90 (70; 102)	92 (77; 119)	NS
GGT, IU/L	56 (38; 112)	53 (30; 116)	58 (46; 112)	NS
Total Bilirubin, mg/dL	0.89 (0.62; 1.20)	0.7 (0.53; 0.82)	1.1 (0.8; 1.5)	NS
Albumin, g/dL	4.1 (3.8; 4.4)	4.3 (4.1; 4.5)	4 (3.7; 4.3)	NS
Platelet Count, ×10^3^ cell/mm^3^	123 (98; 171)	171 (131; 215)	109 (81; 132)	*p* = 0.003
Creatinine, mg/dL	0.83 (0.74; 0.93)	0.83 (0.66; 0.91)	0.85 (0.76; 0.97)	NS
INR	1.1 (1.03; 1.12)	1.04 (1.01; 1.07)	1.09 (1.03; 1.14)	NS
AFP ng/mL	4.4 (2.6; 6.9)	4.8 (3; 23)	4.1 (2.3; 5.3)	NS
PLR	98.5 (86; 133.5)	101 (87.5; 139)	96 (85; 127)	NS
LMR	2.9 (2.1; 3.8)	2.9 (2.5; 4.3)	2.8 (1.95; 3.4)	NS
NLR	2.3 (1.8; 3.2)	2.3 (1.7; 3.2)	2.3 (1.85; 3.4)	NS
Child–Pugh				
A5 A6 A7	44 (75.9%) 13 (22.4%) 1 (1.7%)	18 (78.3%) 5 (21.7%) 0 (0%)	26 (74.3%) 8 (22.9%) 1 (2.8)	NS
MELD	7 (7; 9)	7 (7; 7)	8 (7; 10)	NS
Patients with HCC Recurrence	24 (41.4%)	8 (34.8%)	16 (45.7%)	NS
Recurrence Time from Treatment, months	6 (1; 9)	10.5 (4.75; 12)	2 (1; 6)	*p* = 0.016

**Table 2:** Clinical, biochemical, and staging characteristics of the enrolled population (*n* = 58), categorized according to HCC recurrence. Abbreviations: BCLC: Barcelona Clinic Liver Cancer; AST: aspartate aminotransferase; ALT: alanine aminotransferase; GGT: γ-glutamyl transferase; ALP: alkaline phosphatase; INR: international normalized ration; AFP: α-fetoprotein; PLR: platelet/lymphocyte ratio; LMR: lymphocytes/monocytes ratio; NLR: neutrophils/lymphocytes ratio; NS: not significant; NA: not applicable.

Variables	Altogether N = 58	Without HCC Recurrence N = 34	HCC Recurrence N = 24	Significance
Gender, Male	48 (82.8%)	26 (76.5%)	22 (91.7%)	*p* = 0.032
Age, years	70 (65; 77)	70 (65; 78)	70 (65; 76)	NS
Etiology, Viral	27 (46.6%)	14 (41.2%)	13 (54.2%)	NS
Presence of Liver Cirrhosis	51 (87.9%)	28 (82.4%)	23 (67.6%)	NS
Number of Nodules, number	1 (55, 94.2%) 2 (3, 6.8%)	1 (33, 97%) 2 (1, 3%)	1 (22, 91.7%) 2 (2, 8.3%)	NS
Nodule Maximum Diameter, mm	22 (15; 30)	18 (13; 23.5)	33 (24; 48)	*p* < 0.0001
BCLC				
Stage 0 Stage A	28 (48.3%) 30 (51.7%)	17 (50%) 17 (50%)	11 (45.8%) 13 (54.2%)	NS
AST, IU/L	25.5 (22; 37)	25 (22; 36)	28 (21; 65)	NS
ALT, IU/L	22 (17; 35)	20.5 (15; 33)	25 (18; 59)	NS
ALP, IU/L	92 (75; 112)	94 (77; 114)	84 (75; 101)	NS
GGT, IU/L	56 (38; 112)	50 (30; 111)	60 (48.5; 128)	NS
Total Bilirubin, mg/dL	0.89 (0.62; 1.20)	0.75 (0.58; 1.15)	0.93 (0.75; 1.21)	NS
Albumin, g/dL	4.1 (3.8; 4.4)	4.2 (3.82; 4.4)	4.1 (3.8; 4.5)	NS
Platelet Count, × 10^3^ cell/mm^3^	123 (98; 171)	143 (107; 205)	111.5 (92; 129)	*p* = 0.03
Creatinine, mg/dL	0.83 (0.74; 0.93)	0.87 (0.78; 0.94)	0.80 (0.71; 0.92)	NS
INR	1.1 (1.03; 1.12)	1.05 (1; 1.11)	1.08 (1.03; 1.13)	NS
AFP, ng/mL	4.4 (2.6; 6.9)	4 (2.5; 6.35)	4.7 (3.55; 7.37)	NS
PLR	98.5 (86; 133.5)	103.5 (89.5; 148)	92 (78.5; 114)	*p* = 0.026
LMR	2.9 (2.1; 3.8)	2.6 (2; 3.7)	3.2 (2.7; 4.2)	*p* = 0.041
NLR	2.3 (1.8; 3.2)	2.6 (1.9; 3.4)	1.9 (1.5; 2.8)	*p* = 0.043
Child–Pugh				
A5 A6 A7	44 (75.9%) 13 (22.4%) 1 (1.7%)	24 (70.6%) 10 (29.4%) 0 (0%)	20 (83.3%) 3 (12.5%) 1 (4.2%)	NA
MELD	7 (7; 9)	7 (7; 10)	7 (7; 9)	NS

**Table 3:** Cox regression analysis involving statistically significant different variables between patients with and without HCC recurrence. Abbreviations: PLR: platelet/lymphocyte ratio; LMR: lymphocytes/monocytes ratio; NLR: neutrophils/lymphocytes ratio.

Variable of Interest	Univariate Analysis		Multivariate Analysis	
	Hazard Ratio (95% Confidence Interval)	Significance	Hazard Ratio (95% Confidence Interval)	Significance
Gender, Male	2.5 (1.9–3.1)	*p* = 0.001	3.1 (2.1–3.2)	*p* = 0.001
Nodule Max Diameter > 20 mm	4.5 (3.9–5.1)	*p* < 0.001	5.9 (2.8–6.9)	*p* = 0.001
Nodule Max Diameter > 30 mm	5.1 (4.5–5.5)	*p* < 0.001		
Nodule Max Diameter > 40 mm	6.1 (4.9–6.7)	*p* < 0.001		
Platelet Count < 125 × 10^3^ cell/mm^3^	1.6 (1.2–1.9)	*p* = 0.030		
PLR < 95	2.1 (1.7–2.6)	*p* = 0.022		
LMR < 2.5	1.9 (1.4–2.5)	*p* = 0.019		
NLR > 2	2.7 (2.2–3.3)	*p* = 0.002		

## Data Availability

The data that support the findings of this study are available from the corresponding author upon reasonable request.
